# VEGF-121 plasma level as biomarker for response to anti-angiogenetic therapy in recurrent glioblastoma

**DOI:** 10.1186/s12885-018-4442-2

**Published:** 2018-05-10

**Authors:** Maurizio Martini, Ivana de Pascalis, Quintino Giorgio D’Alessandris, Vincenzo Fiorentino, Francesco Pierconti, Hany El-Sayed Marei, Lucia Ricci-Vitiani, Roberto Pallini, Luigi Maria Larocca

**Affiliations:** 1 Polo Scienze Oncologiche ed Ematologiche, Istituto di Anatomia Patologica, Università Cattolica del Sacro Cuore, Fondazione Policlinico Universitario Agostino Gemelli, Largo Francesco Vito 1, 00168 Rome, Italy; 2 Polo Scienze dell’invecchiamento, Neurologiche, Ortopediche e della Testa-Collo, Istituto di Neurochirurgia, Università Cattolica del Sacro Cuore, Fondazione Policlinico Universitario Agostino Gemelli, Largo Francesco Vito 1, 00168 Rome, Italy; 3 Department of Cytology and Histology, Qatar University, Doha, Qatar; 4 Department of Hematology, Oncology and Molecular Medicine, Istituto Superiore di Sanità, Viale Regina Elena 299, Rome, 00161 Italy

**Keywords:** Recurrent glioblastoma, Antiangiogenetic-therapy, VEGF isoforms, Target therapy

## Abstract

**Background:**

Vascular endothelial growth factor (VEGF) isoforms, particularly the diffusible VEGF-121, could play a major role in the response of recurrent glioblastoma (GB) to anti-angiogenetic treatment with bevacizumab. We hypothesized that circulating VEGF-121 may reduce the amount of bevacizumab available to target the heavier isoforms of VEGF, which are the most clinically relevant.

**Methods:**

We assessed the plasma level of VEGF-121 in a brain xenograft model, in human healthy controls, and in patients suffering from recurrent GB before and after bevacizumab treatment. Data were matched with patients’ clinical outcome.

**Results:**

In athymic rats with U87MG brain xenografts, the level of plasma VEGF-121 relates with tumor volume and it significantly decreases after iv infusion of bevacizumab. Patients with recurrent GB show higher plasma VEGF-121 than healthy controls (*p* = 0.0002) and treatment with bevacizumab remarkably reduced the expression of VEGF-121 in plasma of these patients (*p* = 0.0002). Higher plasma level of VEGF-121 was significantly associated to worse PFS and OS (*p* = 0.0295 and *p* = 0.0246, respectively).

**Conclusions:**

Quantitative analysis of VEGF-121 isoform in the plasma of patients with recurrent GB could be a promising predictor of response to anti-angiogenetic treatment.

**Electronic supplementary material:**

The online version of this article (10.1186/s12885-018-4442-2) contains supplementary material, which is available to authorized users.

## Background

Glioblastoma (GB) is one of the most vascularized human tumors and the abnormal microvascular proliferation, in particular of the so-called glomeruloid vessels, represents a histopathological hallmark of this neoplasia [1, 2]. Hypoxia is a major driving force of this process that determines a consistent upregulation of several proangiogenic factors [3]. Among them, vascular endothelial growth factor-A (VEGF-A, commonly referred to as VEGF) seems to be the most important one, mainly operating in the activation of quiescent endothelial cells and promoting cell migration and proliferation [2–5].

As GBs are highly vascularized cancers with high levels of VEGF, therapies that target angiogenesis have generated substantial interest [6]. In this regard, a humanized anti-VEGF monoclonal antibody, called bevacizumab, has recently been approved for the therapy of recurrent GB [6–9]. However, the initial optimism generated by the therapeutic results in the recurrent setting was tempered by recent Phase III trials showing no efficacy for treating newly diagnosed GBs [6, 10, 11]. This data, together with the clinical evidence that a significant percentage of GBs treated with bevacizumab for an extended period of time undergoes transformation to a more biologically aggressive tumor, leads to uncertainty about the appropriate indications for the use of bevacizumab in GB [12, 13]. Despite these concerns, there remain numerous examples of radiological and clinical improvement after anti-angiogenetic treatment in de novo GB and particularly in patients with recurrent GB with limited therapeutic options. For this reason, the search for predictive biomarkers able to identify those patients who will likely benefit from bevacizumab is a primary focus in the assessment of antiangiogenic therapy for GB [12, 14, 15].

VEGF exists in several isoforms with different molecular weights and biological properties. Heavier isoforms (VEGF-206, VEGF-189) are bound to the extracellular matrix and represent a reserve of VEGF [16, 17]. The intermediate-weight VEGF-165 isoform has an optimal bioavailability and high mitogenic potential. On the contrary, the lighter VEGF-121 isoform, the main one present in circulating blood, has low mitogenic potential and probably plays a minor role in tumor angiogenesis [16–18].

We have recently shown that GB is able to produce all VEGF isoforms and that its sensitivity to bevacizumab may depend on the relative amount of the various isoforms [19]. As bevacizumab binds to all VEGF isoforms, we postulated that in patients with low levels of circulating VEGF-121 a greater amount of bevacizumab may be available to target the heavier and intermediate isoforms of VEGF, which are the most clinically relevant [19, 20].

In the present study, we used a brain xenograft model of human GB cells to demonstrate that the VEGF-121 isoform can be readily detectable in the peripheral blood, that its plasma levels relate with tumor size, and that circulating VEGF-121 significantly decreases after bevacizumab infusion. Then, we analyzed a group of patients with recurrent GB under treatment with anti-angiogenic therapy and showed a significant reduction of plasma VEGF-121 after bevacizumab infusion. Notably, patients with baseline lower levels of VEGF-121 and lower reduction of VEGF-121 after anti-angiogenetic drug infusion showed a better clinical outcome suggesting that levels of circulating VEGF-121 could represent a useful biomarker to predict the efficacy of bevacizumab in GB patients.

## Methods

### Intracranial xenografting of human GB cells in athymic rats and blood sampling

Experiments involving animals were approved by the Ethical Committee of the Università Cattolica Sacro Cuore (UCSC), Rome (Pr. No. CESA/P/51/2012). Immunosuppressed athymic rats (n. 10; male, 250-280 g; Charles River, Milan, Italy) were anesthetized with intraperitoneal injection of diazepam (2 mg/100 g) followed by intramuscular injection of ketamine (4 mg/100 g). Animal skulls were immobilized in a stereotactic head frame and a burr hole was made 3 mm right of the midline and 1 mm anterior to the bregma. The tip of a 10 μl-Hamilton microsyringe was placed at a depth of 5 mm from the dura and 2 × 10^4^ U87MG cells were slowly injected. After grafting, the animals were kept under pathogen-free conditions in positive-pressure cabinets (Tecniplast Gazzada, Varese, Italy) and observed daily for neurological signs. Beginning 4 days after implantation, the rats were treated with bevacizumab (10 mg/kg ip) twice weekly. Control animals were treated with equal volumes of saline. After 28 days of survival, the rats were deeply anesthetized. The aorta was transcardially cannulated and 1.5 ml of blood was taken into a syringe with EDTA as anticoagulant. Then, rats were perfused with saline followed by 4% paraformaldehyde in 0.1 M PBS. The brain was removed, stored in 30% sucrose buffer overnight at 4 °C, and serially cryotomed at 40 μm on the coronal plane. Sections were collected in distilled water, mounted on slides, and stained with cresyl violet. Tumor volumes (in 8 rats) were calculated on histological sections through the tumor epicenter, according to the equation: *V = (a*^*2*^ *x b)/2*, where *a* is the shortest diameter and *b* is the longest diameter of tumors.

### Patients and bevacizumab treatment

The study was conducted on three groups of patients. The first group (*n*, 6) was composed of patients suffering from recurrent GB after having undergone surgery and standard-of-care chemo-radiotherapy (Stupp protocol) [21], who were not eligible for reoperation and received bevacizumab therapy (5 men and 1 woman, aged 45 to 66 years at the time of primary surgery, median age of 55.5 years). The second group (*n*, 6) was composed of patients that completed the Stupp protocol, who showed recurrent tumor on follow-up Magnetic Resonance Imaging (MRI), who were judged eligible for reoperation and did not receive bevacizumab (4 men and 2 women, aged 48 to 76 years at the time of primary surgery, a median age of 59.6 years) (see Table 1). The third group was composed of 10 healthy volunteers who did not receive bevacizumab (7 men and 3 women, aged 50 to 73 years at the time of the analysis, median age of 58.2 years). Treatment of the first group involved the administration of bevacizumab at the dose of 10 mg/kg iv every 2 weeks in 6-week cycles. Immediately before and 30 min after the end of bevacizumab infusion, plasma samples were collected for VEGF-121 analysis. All patients provided written informed consent according to the research proposals approved by the Ethical Committee of the UCSC. Response to treatment was classified using RANO criteria [19]. In each patient, the contrast enhancing tumor (CE) area was calculated on follow-up gadolinium-enhanced T1-weighted MRI in the axial, coronal, and sagittal planes using ImageJ 1.45S software (Rasband, W.S., ImageJ, US NIH, Bethesda, Maryland, USA, https://imagej.net/, 1997-201). Progression-free survival (PFS) and overall survival (OS) were defined as the time between bevacizumab treatment initiation and, respectively, first documentation of progression or death from any cause.

**Table 1:** Patients’ characteristics and clinical features

Patient	Tumor location	*N* surgeries pre-bev	*n* bev cycles	Best response	Toxicity (grade)	PFS (mos)	OS (mos)
1	R temporal	3	11	CR	none	51	71
2	R parietal	1	12	CR	none	36	41
3	R temporal	1	1	PD	brain hemorrhage	3	4
4	R parietal	2	2	SD	hepatic failure	6	8
5	L temporal	1	9	CR	none	40	48
6	multifocal	1	2	PD	brain hemorrhage	6	7
7	L occipital	1	NA	NA	NA	NA	NA
8	multifocal	1	NA	NA	NA	NA	NA
9	L parietal	2	NA	NA	NA	NA	NA
10	R parietal	1	NA	NA	NA	NA	NA
11	R temporal	1	NA	NA	NA	NA	NA
12	L parietal	1	NA	NA	NA	NA	NA

### Enzyme-linked immunosorbent assay

Peripheral blood samples were collected in tube with EDTA as anticoagulant. The plasma samples were centrifuged for 15 min at 1000×g at 4 °C, then plasma was separated and stored in aliquot at − 80 °C until use. Plasma levels of VEGF-121 were quantified using Enzyme-linked immunosorbent assay (ELISA) kit for human-VEGF-121 (SEB851Hu, Cloud-Clone Corp, Huston, TX) according to the manufacturer’s instruction. Quantification was performed spectrophotometrically using LD400, Beckman Coulter (Fullerton, CA) at wavelength of 450 nm. The concentration of VEGF-121 was determined by comparing the optical density (OD) of the samples to the standard curve. The minimum detectable level of VEGF-121 of this kit is typically less than 6.7 pg/ml.

### VEGF-121 mRNA expression in primary GB

The expression of VEGF-121 mRNA was performed as previously described on cultured T98G, U251, and U87MG GB cell lines as well as on the tumor tissue of patients enrolled in this study [19].

### Statistical analysis

Statistical analysis was described in Additional file 1.

## Results

### Plasma VEGF-121 in rats with intracranial xenografts of human U87MG cells

**Fig. 1:**
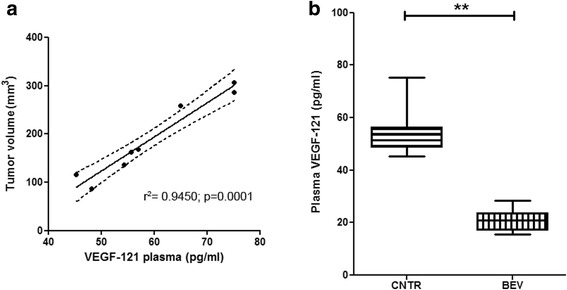
**a** The panel shows the significant correlation between the size of tumor and the VEGF-121 plasma level in the xenografts (linear regression, r^2^ = 0.9450; *p* = 0.0001); **b**. The panel shows the significant reduction of the human VEGF-121 plasma level in rats harboring intracranial xenografts of human GB U87MG cell line, between controls and bevacizumab-treated animals (*p* = 0.0004 Mann-Whitney t test t)

Recently, we found that GB produces different VEGF isoforms and that the clinical and radiological response to bevacizumab is associated with low expression of VEGF-121 mRNA by the tumor tissue [19]. In order to test the hypothesis that antigen-antibody reactions between circulating VEGF-121 protein and infused bevacizumab might reduce the bioavailability of bevacizumab for the heavier VEGF isoforms, we grafted human U87MG cells onto the brain of athymic rats and measured VEGF-121 protein levels in the rat plasma. We used U87cell line for xenograft experiments because this cell line expresses several VEGF isoforms and, in comparison to other glioma cell line, highest level of VEGF-121 (data not shown) [22]. Human VEGF-121 protein was not detectable in the plasma of normal control rats. In rats with U87MG brain xenografts, however, plasma VEGF-121 protein was 55.158 ± 38.38 pg/ml (mean ± sd). The level of VEGF-121 protein in plasma related significantly with the size of tumor xenografts (linear regression, r^2^ = 0.9450; *p* = 0.0001; Fig. 1a). Importantly, after injection of bevacizumab in the tail vein of rats with U87MG brain xenografts, the level of plasma VEGF-121 protein decreased to 20.918 ± 2.32 pg/ml (*p* = 0.0004 Mann-Whitney t test; Fig. 1b). Then, this experiment demonstrated that VEGF-121 protein can be measured in plasma and that its level decreases significantly after infusion of bevacizumab.

### Expression of plasma VEGF-121 protein in patients with recurrent GB

**Fig. 2:**
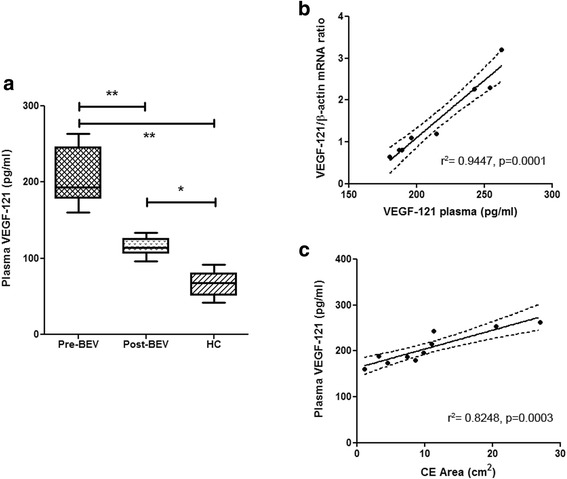
**a**. The figure shows the significantly higher expression of VEGF-121 in the plasma of patients with recurrent GB (Pre-BEV) in comparison to the healthy patients (HC) (*p* = 0.0002, Mann-Whitney t test). After bevacizumab treatment (Post-BEV) patients with recurrent GB showed a significant reduction of the human VEGF-121 plasma level (p = 0.0002, Mann-Whitney t test); **b**. The figure shows the significant correlation between plasma level of VEGF-121 and cancer tissue VEGF-121 mRNA expression (linear regression, r^2^ = 0.9447, *p* = 0.0001); **c.** The figure shows the significant correlation between plasma level of VEGF-121 and contrast enhancing tumor area (linear regression, r^2^ = 0.8248, *p* = 0.0003)

**Fig. 3:**
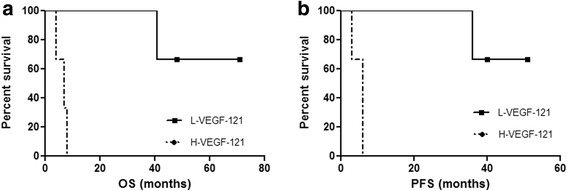
Kaplan-Meier survival curves of patients stratified by VEGF-121 plasma level in patients with recurrent GB after treatment with bevacizumab methylation status. The lower level of VEGF-121 (L-VEGF-121) are significantly associated with a favorable survival advantage in term of OS (**a**; *p* = 0.0246; HR 15.34; 95% CI from 1.418 to 166.0) and PFS (**b**; *p* = 0.0295; HR 16.23; 95% CI from 1.320 to 199.6) in comparison with those recurrent GBs with higher level (H-VEGF-121)

We first assessed VEGF-121 protein level in plasma of healthy volunteers (*n*, 10), where we detected values of 66.789 ± 17.431 pg/ml (mean ± sd). In plasma of patients with recurrent GB (*n*, 12), however, the level of this isoform was about three folds higher (206.321 ± 35.693 pg/ml; *p* = 0.0002; Mann-Whitney t test; Fig. 2a). Moreover, patients with higher plasma level of VEGF-121 also had higher expression of mRNA of this isoform in the tumor tissue obtained at surgery with a significant relationship between the two variables (linear regression, r^2^ = 0.9447, *p* = 0.0001; Fig. 2b). After iv infusion of bevacizumab, the level of VEGF-121 in the plasma of GB patients lowered (*n*, 6; 115.076 ± 12.746 pg/ml) with a significant reduction in comparison to pre-infusion level (*p* = 0.0002 Mann-Whitney t test; Fig. 2a). Despite its drop after iv bevacizumab, VEGF-121 plasma level remained significantly higher than healthy volunteers (*p* = 0.0022 Mann-Whitney t test; Fig. 2a). Interestingly, when we correlated the contrast enhancing (CE) tumor area with the VEGF-121 plasma level measured before infusion of bevacizumab, we found a linear correlation where tumors with larger CE area showed higher plasma level of VEGF-121 (linear regression, r^2^ = 0.8248, *p* = 0.0003; Fig. 2c). When we compare recurrent GB patients with higher VEGF-121 plasma level before the bevacizumab treatment (greater than the median value > 211.735 pg/ml) with patients with lower level of VEGF-121 (lower that the median value), we found a significant association between lower level of this VEGF isoform and a better prognosis (OS, *p* = 0.0246; HR 15.34; 95% CI from 1.418 to 166.0; PFS, *p* = 0.0295; HR 16.23; 95% CI from 1.320 to 199.6; Fig. 3). Finally, by relating PFS and OS either to baseline VEGF-121 plasma level or to differential VEGF-121 (ΔVEGF121 = VEGF-121 level at baseline – VEGF-121 level after bevacizumab infusion), we observed that higher level of baseline VEGF-121 and higher ΔVEGF121 were significantly associated with worse PFS and OS (*p* = 0.0001 and 0.0003, and *p* = 0.0013 and 0.0008, respectively; linear regression test; Additional file 2: Figure S1).

## Discussion

In the search for molecular mechanisms that may underlie the response of recurrent GB to anti-VEGF treatment, we have recently found that this tumor is able to produce different VEGF isoforms and that better clinical responses to bevacizumab are significantly associated with low levels of VEGF-121 mRNA in the tumor [19]. We hypothesized that this circulating isoform of VEGF could interfere with the availability of bevacizumab in neutralizing heavier and intermediate isoforms of VEGF, which play a major role in brain tumor angiogenesis [19, 22]. Here, we showed that the human VEFG-121 isoform can be detected in plasma of rats harboring intracranial graft of human U87MG GB cells, and that following iv infusion of bevacizumab plasma VEGF-121 is significantly lowered. In patients with recurrent GB, we also demonstrated a significant association between level of VEGF-121 mRNA in the tumor and VEGF-121 protein level in plasma. Indeed, these patients have three-fold higher level of plasma VEGF-121 protein compared to healthy controls. Consistent with the in vivo findings, VEGF-121 plasma level significantly decreased after bevacizumab infusion. Our selection criteria for bevacizumab therapy in patients with recurrent GB are quite stringent [23], restricting the size of our patient cohort, thought definitive conclusions cannot drawn and larger series are warrant, this study shows that recurrent GBs with low plasma VEGF-121 or with mild reduction of VEGF-121 level after bevacizumab infusion have a better clinical outcome in terms of PFS and OS.

Although GB produces all isoforms of VEGF [19, 22, 24, 25], the functions of various isoforms and their ability to bind to different types of VEGF receptors in high grade gliomas is still debated. Some evidences highlight that VEGF-165, by virtue of its intermediate extracellular matrix-binding properties, has optimal characteristics of bioavailability and biological potency (higher mitogenic potential), whereas the diffusible VEGF-121 plays a more dynamic role, showing low mitogenic potential [18, 22, 24–26]. In addition, either VEGF-165 and VEGF-189 strongly augment neovascularization, mainly represented by more mature and structured vasculature, probably through the ability of these seven exon encoding isoforms to interact with the co-receptor Neuropilin-1 (Nrp1) and to bind Nrp1-expressing monocytes that, in turn, act in a paracrine manner recruiting smooth muscle cells around the newly formed vessels [24, 26, 27]. Moreover, a recent paper demonstrated that in the tumor interstitium the free VEGF is 7 to 13 times higher than in plasma and that such free VEGF is mostly (> 70%) composed by VEGF-121. This observation reinforces our hypothesis that VEGF-121 may reduce availability of bevacizumab due to antigen-antibody reactions both in circulating blood and in tumor microenvironment.

Our in vivo experiments also demonstrate that VEGF-121 produced by intracerebral GB tumor diffuses along the tumor interstitium crossing the altered BBB. In this way, we interestingly found a significant association between VEGF-121 plasma levels and tumor volume in xenograft and CE area in recurrent GB before infusion of bevacizumab. Although the prognostic value of the tumor volume and the CE area in high-grade gliomas is highly controversial [28, 29], the correlation between diffusible VEGF-121 isoform plasma level and these parameters might be related to a higher cancerous angiogenesis and probably to a greater breakdown of the BBB that would favor the plasma transfer of this isoform.

This data suggests that quantitative testing of plasma VEGF-121 could be useful in patients’ selection for bevacizumab therapy.

## Conclusions

To conclude, our results clearly indicate that VEGF-121 isoform plasma level is a biomarker for GB tumors and that it may predict the response to anti-angiogenetic treatment. The predictive power of baseline VEGF-121 in the plasma and the drop of this isoform level after bevacizumab infusion need to be validated by larger and multicenter clinical studies. At the same time, our results pave the way for the development of novel therapeutic approaches where a more selective anti-VEGF-165 antibody might lead to an increased efficacy of anti-angiogenetic therapy.

## Additional file

Additional file 1: Statistical analysis. (DOCX 13 kb)

Additional file 2: **Figure S1.** Panels A and B. The panels show the significant correlation between plasma level of VEGF-121 and, respectively, OS (panel A; linear regression test: *p* = 0.0013; r^2^ = 0,9417), and PFS (panel B; linear regression test: *p* = 0.0001; r^2^ = 0,9913). Panels C and D. The panels show the significant correlation between differential plasma value of VEGF-121 (∆VEGF121: VEGF-121 level at baseline – VEGF-121 level after bevacizumab infusion) and, respectively, OS (panel C; linear regression test: *p* = 0.0008; r^2^ = 0,9731), and PFS (panel D; linear regression test: *p* = 0.0003; r^2^ = 0,9742). (TIF 1478 kb)

## Abbreviations

ELISA: Enzyme-linked immunosorbent assay
GB: Glioblastoma
iv: Intravenous infusion
MRI: Magnetic resonance imaging
Nrp1: Neuropilin-1
OD: Optical density
OS: Overall survival
PFS: Progression-free survival
SD: Standard deviation
VEGF: Vascular endothelial growth factor
