# Supplementary material for: VEGF-121 plasma level as biomarker for response to anti-angiogenetic therapy in recurrent glioblastoma
Source: BMC Cancer. 2018 May 10;18:553. doi: 10.1186/s12885-018-4442-2 (PMC5946426; doi:10.1186/s12885-018-4442-2)
Supplement: Supplementary file 1 — Statistical analysis. (DOCX 13 kb) [file 12885_2018_4442_MOESM1_ESM.docx]

**Supplemental data**

**Statistical analysis**

Statistical analysis was performed using GraphPad Prism (version 5, La Jolla, CA) or MedCalc (version 10.2, Ostend, Belgium) software. Continuous variables normally distributed are reported as mean and standard deviation (SD). Comparison of continuous variables was performed using the Student’s t test (non-parametric Mann-Whitney t test). Comparison of categorical variables between the two groups was performed by the Chi-square statistic, using the Fisher exact test when appropriate. A linear regression analysis was performed to evaluate the significant association between different variables. Kaplan-Meier survival curves were plotted and differences in survival between groups of patients were compared using the log-rank test. All *p*-values are considered statistically significant when *p*<0.05. Asterisks indicate the level of statistical significance (**p* < 0.05; ***p* < 0.001).
