# Supplementary figures and images for: VEGF-121 plasma level as biomarker for response to anti-angiogenetic therapy in recurrent glioblastoma
Source: BMC Cancer. 2018 May 10;18:553. doi: 10.1186/s12885-018-4442-2 (PMC5946426; doi:10.1186/s12885-018-4442-2)

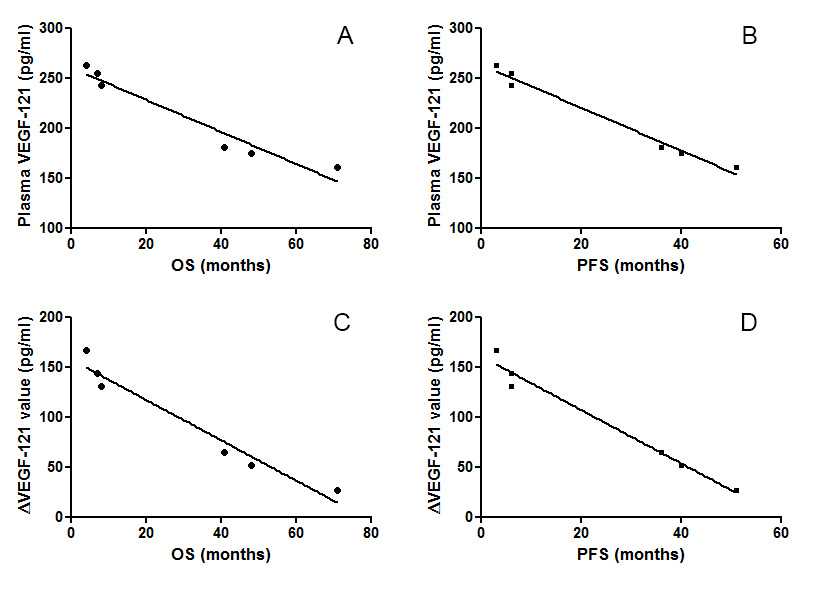

Supplement: Supplementary file 2 — Figure S1. Panels A and B. The panels show the significant correlation between plasma level of VEGF-121 and, respectively, OS (panel A; linear regression test: p = 0.0013; r2 = 0,9417), and PFS (panel B; linear regression test: p = 0.0001; r2 = 0,9913). Panels C and D. The panels show the significant correlation between differential plasma value of VEGF-121 (∆VEGF121: VEGF-121 level at baseline – VEGF-121 level after bevacizumab infusion) and, respectively, OS (panel C; linear regression test: p = 0.0008; r2 = 0,9731), and PFS (panel D; linear regression test: p = 0.0003; r2 = 0,9742). (TIF 1478 kb) [file 12885_2018_4442_MOESM2_ESM.tif]
